# Clinical and Biochemical Evaluation of the Use of Alb-PRF versus L-PRF in Mandibular Third Molar Extractions: A Split-Mouth Randomized Clinical Trial

**DOI:** 10.3390/jfb14100505

**Published:** 2023-10-10

**Authors:** Kayvon Javid, Carlos Fernando Mourão, Rafael Coutinho Mello-Machado, Suelen Cristina Sartoretto, Madelaine Torres, Emanuelle Stellet Lourenço, Paulo Emilio Correa Leite, José Mauro Granjeiro, Gutemberg Gomes Alves, Monica Diuana Calasans-Maia

**Affiliations:** 1 Graduate Program in Dentistry, Fluminense Federal University, Niterói 24020-140, Brazil; 2 Department of Periodontology, Tufts University School of Dental Medicine, Boston, MA 02111, USA; 3 Department of Implant Dentistry, Universidade Iguaçu, Nova Iguaçu 26260-045, Brazil; 4 Department of Oral Surgery, Fluminense Federal University, Niterói 24020-140, Brazil; 5 Clinical Research Unit, Antonio Pedro Hospital, Fluminense Federal University, Niterói 24033-900, Brazil; 6 Department of Biotechnology, Fluminense Federal University, Niterói 24033-900, Brazil

**Keywords:** platelet-rich fibrin, albumin, lower third molars, L-PRF, Alb-PRF

## Abstract

Bone tissue engineering seeks biomaterials that enable cell migration, angiogenesis, matrix deposition, and tissue regeneration. Blood concentrates like platelet-rich fibrin (L-PRF) offer a cost-effective source of cells and growth factors to enhance healing. The present study aimed to evaluate heated serum albumin with liquid PRF (Alb-PRF) and L-PRF clinically and biochemically after placement in dental sockets following mandibular third molar extraction. In a controlled, split-mouth study involving 10 volunteers, 20 extracted molars were treated with either Alb-PRF or L-PRF. Post-extraction, pain, trismus, infection presence, and swelling were measured. The concentrations of different analytes in the surgical sites were also examined. The data were statistically analyzed, with significance defined at *p* < 0.05 (*t*-test). No significant difference was noted between the groups for pain and trismus, but Alb-PRF showed a significant reduction in swelling on day seven. The Alb-PRF group showed lower levels of pro-inflammatory cytokines (GM-CSF, IL-1b, IL-6, IFNy, IL-8, IL-15, RANTES, and MIP-1a) after seven days, with only higher expressions of MIP-1b, IL-1b, and MCP-1 found in the L-PRF group. Differences were observed in the release of analytes between L-PRF and Alb-PRF, with Alb-PRF significantly reducing edema after seven days. Alb-PRF reduced edema, while L-PRF increased inflammatory cytokines. When compared to L-PRF, Alb-PRF reduced edema and the release of inflammatory cytokines, suggesting promising effects in socket healing while underscoring the role of growth factors and cytokines in potential applications of blood concentrates.

## 1. Introduction

Bone tissue, a specialized connective tissue, exhibits a substantial capacity for regeneration and remodeling, which is crucial for maintaining structural and functional integrity [1]. Minor fractures often heal without leaving fibrous scar tissue [2,3,4]. However, localized bone loss resulting from infections, tooth extraction, fractures, surgical resections, or under certain pathological conditions like vascular compromise or metabolic disorders may lead to fibrous tissue formation [5,6], disrupting functionality and aesthetics and impacting overall quality of life.

To address the challenges in bone tissue bioengineering, researchers have focused on developing new biomaterials that can serve as three-dimensional scaffolds. These innovative biomaterials enable cell migration, angiogenesis, new extracellular matrix deposition, mineralization, and tissue regeneration. Ideally, bone substitute biomaterials should include molecules that promote bone differentiation, but this can be costly.

Blood concentrates offer a clinically relevant and cost-effective alternative as a source of cells and growth factors [7]. These concentrates, derived from the patient’s peripheral blood through multiple centrifugation protocols, release various growth factors to augment the healing [8]. One of the byproducts of blood is called leukocyte platelet-rich fibrin (L-PRF). This autologous biomaterial can be obtained through a simple technique that involves a single centrifugation process without any anticoagulant additives [8,9,10]. The presence of platelets and leukocytes allows for the continuous production and release of various growth factors, making it a cost-effective option [11]. Previous research has documented the effectiveness of L-PRF in various clinical applications, such as controlling hemostasis in oral procedures [12,13] and treating osteonecrosis of the jaws [14,15].

The L-PRF is a structure that comprises fibrin, platelets, leukocytes, and plasma proteins. It is often utilized as a safeguarding layer for soft tissues and for techniques that require guided bone regeneration. However, the L-PRF membrane is not suitable for procedures that need prolonged protective barriers due to its high bioabsorption and reduced stability. Although the exact residency time of the membrane post-surgery [14,16,17] remains uncertain, it has been shown that it can actively release cytokines for up to 28 days in a biological medium [18]. However, after this period, the membrane was found to be partially degraded. It’s important to note that this was an in vitro study and did not involve enzymes that could degrade the L-PRF.

To overcome their stability limitations, a new process was introduced that involves adding the liquid portion of L-PRF to denatured platelet-poor plasma. Initially called Alb-CGF (albumin with the presence of concentrate of growth factors) [19], the process was later renamed Alb-PRF (albumin with liquid PRF) in subsequent articles by authors from the same group [20,21]. This process results in the formation of a malleable membrane made up of dense protein structures encased in fibrin fibers that trap cells and platelets. It has demonstrated impressive structural stability for 21 days in mice subcutaneous tissue [21], along with the capability for gradual cytokine and growth factor release [19,21]. Furthermore, albumin’s proven capacity to carry various drugs due to interactions with its three specific domains makes it a promising candidate for drug delivery functions [22,23,24].

The use of albumin in tissue engineering has been extensively documented [25]. This is due to its abundance, ease of isolation from blood plasma precipitation, high purity, and homogeneity [26,27]. Albumin-enriched biomaterials provide an optimal structure for cell proliferation and show minimal reduction over time, suggesting less in vitro degradation [25]. Studies further suggest that the association with albumin can stabilize the fibrin network’s ultrastructure [27]. In addition, preliminary data indicate that combining denatured serum albumin significantly enhances the PRF-based scaffold, yielding an autologous, biocompatible material with the potential for enhanced durability and sustained action [20,21,24]. 

The present study performed lower third molar extraction as a clinical model for assessing wound healing and evaluating the autologous biomaterials. This common procedure is often used to assess the efficacy of blood concentrates (e.g., L-PRF) [28,29,30,31]. The studies reported that the most common postoperative complications are pain, swelling, trismus, and infection [26,32,33]. By carefully selecting cases and standardizing procedures, confounding factors in clinical research can be reduced. This approach is essential for ensuring that the results obtained from research are reliable and accurate. Therefore, paying close attention to these aspects is crucial to achieve successful outcomes. Thus, the model using lower third molar extraction enables, for example, a comparison of treatment effects on clinically relevant outcomes like infection rates, healing, and acute inflammatory symptoms [28,30,33,34]. Due to this, mandibular third molar surgery can provide an optimal procedure to clinically evaluate and directly compare the performance of Alb-PRF for socket healing.

The aim of the research was to assess Alb-PRF and L-PRF, both clinically and biochemically, after being placed in dental sockets following the removal of mandibular third molars.

## 2. Materials and Methods

### 2.1. Ethical Considerations

This study was a randomized, controlled, double-blind, split-mouth study. It was conducted according to the principles described in the Helsinki Declaration regarding experiments on human beings and following Normative Resolution n.466 of 2012 of the National Health Council (CNS), from the Brazilian Ministry of Health. The Ethics Committee approved this study (no. 5,072,786). In addition, this study followed the CONSORT-statement guidelines [35] to ensure the present randomized study’s quality and transparency. Subject volunteers were recruited after they agreed to participate in the study and signed an informed consent form (ICF) agreeing to follow the proposed guidelines and schedule. The sample consisted of 22 post-extraction sockets (11 subjects). All teeth with no treatment possibilities, as verified via clinical and radiological examination by another professional not involved in the study, were recommended for extraction.

### 2.2. Eligibility Criteria

The present clinical trial was designed to encompass a specific demographic group which, upon meeting certain criteria, was essential to the validity of the study. The following criteria outlined the ideal profile of a prospective participant.

The study targeted individuals who were aged over 18 and who exhibited an indication of mandibular third molar exodontia. This cohort should have either erupted or partially erupted Class 1A and 1B according to Pell and Gregory 123 classification for mandibular third molars impaction [36]. It was also required that participants expressed their willingness to cooperate with the study and had already signed the informed consent form. Moreover, a platelet count above 150,000 mm^3^ was a crucial health parameter that needed to be satisfied.

On the other hand, the trial also defined a strict set of exclusion criteria. These criteria were established to control the variable factors that could have interfered with the results of the study. For instance, participants who had mandibular third molars that were unerupted, impacted, or in a horizontal, mesio- or distal-angled position were not considered for the study. Additionally, individuals with conditions such as diabetes, carriers of blood dyscrasias, or metabolic bone diseases (including osteomalacia, hypocalcemia, hypercalcemia, and osteoporosis) were also excluded from participation.

Medication usage was another area of concern; individuals using drugs that could have altered or compromised the bone healing response, such as prolonged use of bisphosphonates or corticoids, were deemed ineligible. The same rule applied to those with a history of anxiety, mood, eating, or psychotic disorders, given that these conditions could have affected their ability to participate and collaborate in the study.

Furthermore, any motor dysfunction that inhibited the performance of oral hygiene led to the exclusion of the candidate from the study. Those who smoked, unless they had been without smoking for at least six months, were not eligible. Pregnant women or infants, as well as participants who had undergone radiotherapy, chemotherapy, or any other cancer treatment, also fell into the category of exclusion due to the potential risks these situations could have posed to the study or the individual’s health.

### 2.3. Sample Size Calculation, Randomization, and Blinding

The sample size calculation was performed using PS Power and Sample Size Calculations version 3.1.6 (Vanderbilt University School of Medicine, Nashville, TN, USA). Based on a preliminary evaluation with five subjects, the power analysis, using a two-tailed *t*-test, revealed that a sample size of at least eight subject volunteers in each group would provide 90% power to detect a significant difference between the groups based on the pain evaluation using the Visual Analogue Scale (VAS) at a 5% significance level. Considering a 20% dropout rate, a sample size of 10 subjects per group was determined to be necessary.

The sample size calculation was performed using PS Power and Sample Size Calculations version 3.1.6 (Vanderbilt University School of Medicine, Nashville, TN, USA). The method of randomization of subject volunteers was intra-participant via a coin system (heads and tails). For the present study, the evaluator and the subject volunteers were blinded to the type of platelet concentrate used inside the socket, thus characterizing a double-blind study.

### 2.4. Preparation of Platelet Concentrates

#### 2.4.1. L-PRF Preparation (Control Group)

Initially, blood was collected in two sterile 9 mL red cap tubes without the presence of an anticoagulant (BD Vacutainer^®^, Becton Serum Blood Collection Tubes, Dickinson & Company, Franklin Lakes, NJ, USA) at room temperature, 22 °C. L-PRF membranes were produced using tubes according to the manufacturer’s instructions, with centrifugation at 2700 rpm for 12 min (~708× *g*) using a fixed angle/vertical centrifuge (IntraSpin™, Biohorizons^®^, Birmingham, AL, USA). This centrifugation protocol considers the g-force value referenced to the bottom of the centrifuge tubes (RCF-max) [37,38]. After centrifugation, each L-PRF membrane was removed from the tube and separated from the red phase at the base using sterile forceps.

#### 2.4.2. Alb-PRF Preparation

Blood samples were collected using 9 mL plastic PET tubes (BD Vacutainer^®^, Becton Serum Blood Collection Tubes, Dickinson & Company, Franklin Lakes, NJ, USA). To produce each membrane, two tubes were inserted into a centrifuge (IntraSpin™, Biohorizons^®^, Birmingham, AL, USA), and the protocol for L-PRF was applied to obtain the liquid-phase PRF (plasma + cell-rich portion), namely centrifugation at 2700 rpm for 12 min (~708× *g*) using a fixed angle/vertical centrifuge (IntraSpin™, Biohorizons^®^, Birmingham, Alabama, AL, USA). After processing, it was possible to visualize the plasma and the remaining blood containing red blood cells. Approximately 2 mL of the initial portion of plasma was collected with a syringe of 3 mL and 18 G needle (Injex^®^, São Paulo, Brazil), while the rest of the blood (cell-rich portion and RBCs) was preserved at room temperature (22 °C).

The syringes containing platelet-poor plasma (PPP) were inserted into a device for denaturing human protein plasma-activated plasma albumin gel (APAG^®^, Silfradent, Italy) for 10 min at an operating temperature of 75 °C. After 10 min at a temperature of 70 °C, the syringes were stored at room temperature for another 10 min to allow cooling.

Subsequently, using a 5 mL syringe with an 18 G needle (Injex^®^, São Paulo, Brazil), 4 mL of the rich portion of the buffy coat was collected, added to the heated PPP layer in the glass container, and mixed gently. After the fibrin polymerization, the process was completed in about five minutes with the formation of the membrane.

The procedures used to obtain peripheral blood to produce the L-PRF and Alb-PRF were performed on the day of the surgeries, immediately before the beginning of the dental extractions. Furthermore, five subjects were aleatorily chosen to donate a blood sample to prepare the blood byproducts for assessing the in vitro release of biological mediators.

### 2.5. Surgical Procedures

Medical and dental anamneses were performed on all participants. Initially, the participants were diagnosed and selected based on clinical examination to evaluate the need for dental extractions as confirmed with periapical/panoramic radiography of the face. Intraoral photographs were taken of all participants pre- and post-treatment after they agreed and signed the Informed Consent Form. Site asepsis was performed by rinsing the mouth with 0.12% chlorhexidine digluconate (Periogard^®^ Colgate, New York, NY, USA) for one minute and extraoral with 4% chlorhexidine soap (Riohex Rioquímica^®^, Duque de Caxias, Brazil).

Then, local anesthesia was administered (using Carpule syringe, Dowell^®^, Rancho Cucamonga, CA, USA) for the inferior alveolar, lingual, and buccal nerves using alphacaine 2% with epinephrine 1:100,000 (DFL Indústria e Comércio^®^, Rio de Janeiro, Brazil). The release of the soft tissue around the tooth was performed using scalpel handle #3 (Bard Park, Dowell^®^, Rancho Cucamonga, CA, USA) and blade #15c (Solidor, Lamelid^®^, Osasco, Brazil) to test the success of deep anesthesia and for better apical positioning of the lever and forceps. The detachment of the tissue was performed using the Molt detacher n° 9 (Dowell^®^, Rancho Cucamonga, CA, USA) around the tooth, and then luxation using an elevator and forceps (Dowell^®^, Rancho Cucamonga, CA, USA) for subsequent tooth removal. After finishing the exodontia, the socket was delicately curetted with Lucas curette n° 4 (Dowell^®^, Rancho Cucamonga, CA, USA) and rinsed with 0.9% saline solution. The same dental surgeon performed all the extractions (C.F.M.).

After tooth removal, each socket was filled with L-PRF (G1) or Alb-PRF (G2) according to randomization for each side in the same research participant. Then, the socket was sutured with Johnson 4–0 silk thread (J&J Ethicon^®^, Jardim das Indústrias, São José dos Campos, Brazil) using an “X” stitch.

In both groups, the suture was performed without tension, and the research participants received Azithromycin 500 mg (Astro, Eurofarma Laboratórios S.A.^®^, Itapevi, Brazil) starting on the day of surgery and maintained for four days [32,39]. The research participants were also instructed to perform oral hygiene using Chlorhexidine gel 0.2% (Perioxidin gel, Laboratório Gross S.A., Sao Paulo, Brazil) twice a day, starting on the day of surgery and maintained for 14 days, and analgesia with 500 mg of Paracetamol (Medley^®^, Campinas, Brazil) only in case of pain.

### 2.6. Postoperative Evaluation

One trained evaluator (M.T.) examined all subject volunteers after 7 and 14 days. The consultations were performed by the same examiner and always at the same time. Parameters such as pain, trismus, swelling, presence or absence of infection, and soft tissue healing were evaluated. The pain was analyzed according to the VAS, with 0 being no pain and 10 being the most severe pain, together with the graphic rating scale [28]. The research participants received the printed scale and were instructed on how to fill it in before and after the surgical procedure. The subject volunteers filled out the scale daily during the first seven days after surgery and returned the document at the last appointment for recording and tabulation of the data. The number of analgesics consumed during this period was also recorded.

Trismus was evaluated from the interincisal distance measured from the mesial edges of the upper and lower right incisors during maximum mouth opening, as described by UStün et al. [40]. The presence of infection in the dental sockets was clinically evaluated and recorded on postoperative days 1, 7, and 14. Soft tissue healing evaluation was performed using the Landry index, which classifies the healing process as very poor, poor, good, very good, and excellent according to the presence of granulation tissue, bleeding at the slightest touch, exposure of connective tissue at the margin of the incision, and the presence or absence of suppuration.

The swelling was evaluated using a modification of Gabka and Matsumara’s tape measure method [28,41,42], with pre-and postoperative measurement in the following areas: from the tragus to the pogonion, from the tragus to the labial commissure, and from the external palpebral commissure to the mandible angle. The sum of the three preoperative measurements was used for each side. They were measured again after 7 and 14 days postoperatively, and the difference between the preoperative and postoperative values established the swelling value of the day.

### 2.7. Assessment of the In Vitro Release of Biological Mediators by Alb-PRF and L-PRF Membranes

To compare the ability of cytokine and growth factor release between Alb-PRF and L-PRF membranes, blood samples were prepared as described above and cultured (*n* = 5) for 7 days in 6-well culture plates (TPP^®^, Burlington, MA, USA), in the presence of 4 mL of DMEM (Dulbecco’s Modified Eagle’s Medium, GIBCO, Waltham, MA, USA), without the use of antibiotics, in a humid atmosphere at 37 °C and 5% CO_2_. The conditioned media were collected and stored in a freezer at −80°. A multiparametric immunoassay based on XMap-labeled magnetic microbeads (LuminexCorp., Austin, TX, USA) was employed through a commercial kit (27-plex panel, Biorad Inc., Hercules, CA, USA) capable of quantifying IL-1β, IL-10, IL-12 (p70), IL-13, IL-15, IL-10, IL-10, IL-8, IL-17, CCL11, FGF-b, CSF3, CSF2, IFN-γ, CXCL10, CCL2, CCL3, CCL-4, PDGF, CCL5, TNFα, and VEGF. Quantification of the magnetic beads was performed with a BioPlex MAGPIX system (Biorad Inc., Hercules, CA, USA), and the results were analyzed using Xponent v. 3.0 software (LuminexCorp., Austin, TX, USA).

### 2.8. Evaluation of Cytokines and Growth Factors in the Surgical Sites

On days 1 and 7 after surgery, a swab collection was performed in each operated region of the participants to perform the quantification of cytokines and growth factors present in the site over time, as previously described. The swabs were immersed in 15 mL falcon tubes containing 1.5 mL phosphate-buffered saline solution (PBS) with 0.2% sodium dodecyl sulfate (SDS) and 0.5% propylene glycol and sonicated for 30 min on an ultrasonic bath with ice maintained at 4 °C for protein extraction. The liquid was collected and stored on an ultrafreezer at −80 °C. The cytokines and growth factors from the surgical sites were then detected using the same multiparametric assay described above for the in vitro release of mediators. Quantification was performed with a BioPlex MAGPIX system (Biorad Inc.), and the results were analyzed using Xponent v. 3.0 software (LuminexCorp.).

### 2.9. Statistical Analysis

In the variables swelling, analgesic consumption, and pain (Visual Analog Scale), the data obtained were expressed as mean with a 95% confidence interval. After applying the D’Agostino and Pearson normality test (*p* < 0.05) and removing outliers with the ROUT method (robust regression and outlier removal, Q = 1%), the Tukey test for multiple comparisons (mixed effect analysis) and paired (*p* < 0.05) was applied. Calculations and graphs were performed in Prism 9.0 (GraphPad Software Inc., San Diego, CA, USA).

Also, in the trismus variable, after applying the D’Agostino and Pearson normality test (*p* < 0.05) and removing outliers with the ROUT method (robust regression and outlier removal, Q = 1%), data were expressed with mean and confidence interval. Paired Student’s *t*-test was applied to identify differences between groups (*p* < 0.05).

Data from the cytokine and growth factor assessment for the control and PRF sites were analyzed by nonparametric, paired Mann–Whitney U tests. The correlation between these results and the blood cell counting (platelet and lymphocytes) was investigated through Spearman’s Rank Correlation Test, where only strong correlations (coefficient above 0.7) were considered. For all tests, an alpha error of 5% was considered. The tests were performed with help of the GraphPad Prism 9.0 (GraphPad Software Inc., San Diego, CA, USA).

## 3. Results

The sample of subjects’ volunteers consisted of three men and seven women, with a mean age of 22.1 ± 3.14 years. Of the 20 teeth, 2 had extensive carious lesions, and 16 had a previous history of pericoronitis. Regarding the position of the teeth, 3 presented mesioangular impaction, and 17 with horizontal impaction according to Winter’s classification. Regarding education, one research participant had a complete college degree, and nine had a full high school degree. All surgeries lasted an average of 9.52 ± 1.73 min per site following local anesthesia.

Postoperative follow-up indicated a good recovery in all cases, with no severe complications, and no research participant presented intolerance to the prescribed medications or side effects/adverse effects. 

**Figure 1:**
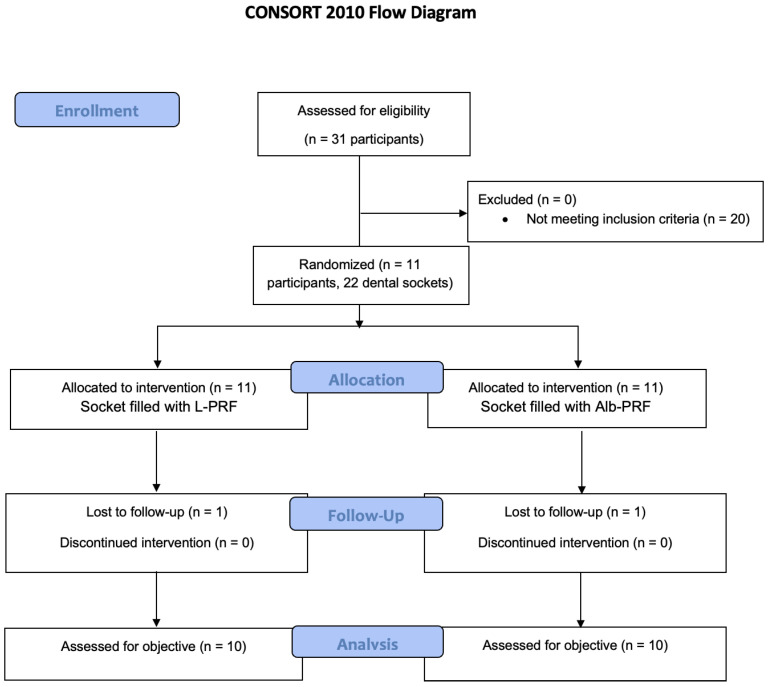
Flowchart of the process of inclusion, allocation, and analysis of the research participants recruited according to CONSORT.

The CONSORT flowchart is shown in Figure 1.

### 3.1. Pain

**Figure 2:**
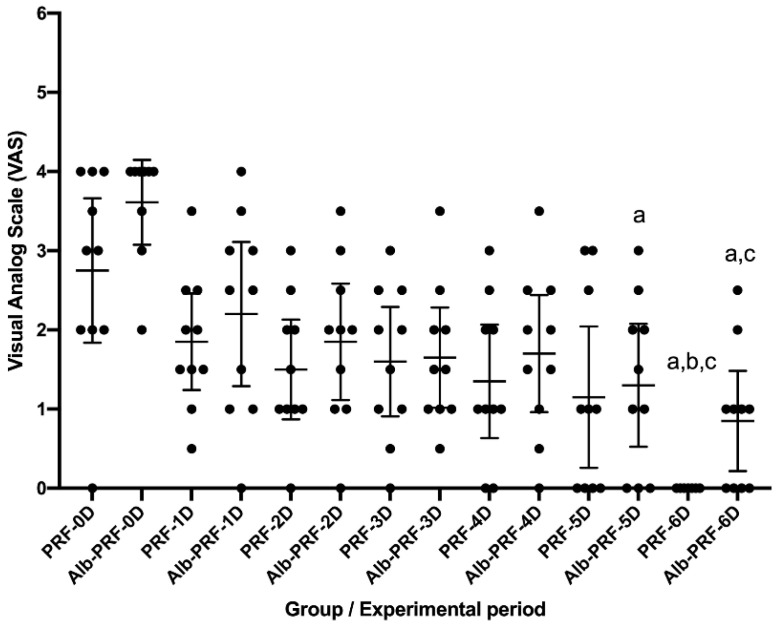
Postoperative evaluation of pain according to the visual analog scale. The graph expresses the mean and confidence interval across points (*n* = 10) on days 0, 1, 2, 3, 4, 5, and 6 for both groups. Comparison between experimental periods identified a significative difference represented by the letters a (≠Day 0 within the same group), b (≠Day 1 within the same group), and c (≠Day 2 within the same group). Multiple Tukey’s tests (mixed effect analysis) and paired comparisons (*p* < 0.05).

No research participant reported difficulty distinguishing pain and its intensity between the right and left sides. Postoperative assessment using the Visual Analog Scale (VAS) was performed on days 0, 1, 2, 3, 4, 5, and 6 in both groups (L-PRF and Alb-PRF). In the Alb-PRF group, days 5 and 6 saw reduced pain according to the VAS compared to day 0. In addition, on day 6, the pain was reduced compared to day 2 (*p* < 0.05). In the L-PRF group, day 6 saw reduced pain compared to days 0, 1, and 2 (*p* < 0.05). No significant difference was observed between the L-PRF and Alb-PRF groups in the same experimental period (*p* < 0.05) (Figure 2).

### 3.2. Swelling

**Figure 3:**
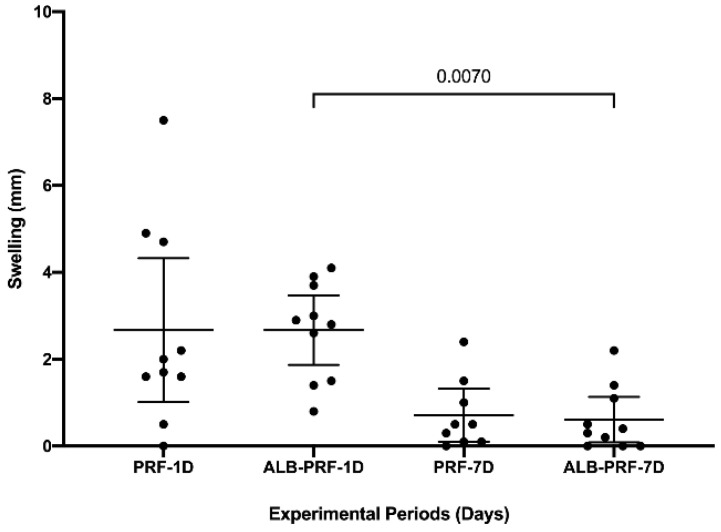
Evaluation of swelling. Graph expressed as mean and confidence interval showing the points (*n* = 10) in the experimental periods of 1 day and 7 days evaluated for the L-PRF and Alb-PRF groups. The comparison between groups did not identify significant differences. The comparison between periods showed a reduction in edema in the Alb-PRF group. Tukey’s test for multiple comparisons (mixed effect analysis) and paired (*p* < 0.05).

All research participants were evaluated on the day of surgery and seven days after for quantification of postoperative edema. All research participants allowed the measurements to be taken without difficulty. Figure 3 shows both groups’ swelling results 1 and 7 days after surgery. The Alb-PRF group showed a significant reduction in edema in the 7-day experimental period (0.61 mm; C.I. 0.08–1.13) when compared to the one-day (2.67 mm; C.I. 1.86–3.47) (*p* = 0.007). There was no statistical difference between the L-PRF and Alb-PRF groups in the periods evaluated (*p* > 0.05).

### 3.3. Trismus

**Figure 4:**
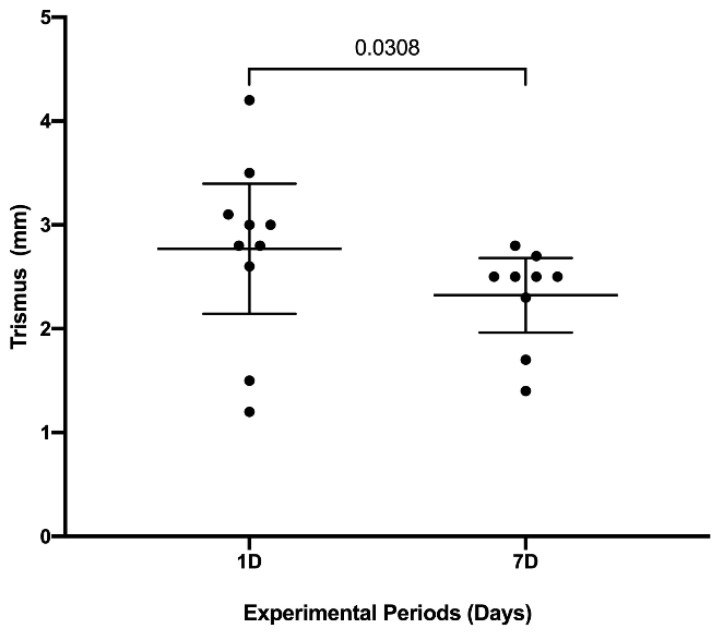
Postoperative trismus assessment 1 and 7 days after surgery. The graph expresses the medians and confidence intervals through the points (*n* = 10). Comparison between experimental periods identifies a significant difference represented by the bar (Paired Student T Test, *p* < 0.05).

Figure 4 shows the results of the postoperative trismus evaluation after 1 and 7 days. There was a significant reduction in mouth opening limitation after seven days of surgery (2.32; C.I. 1.96–2.68) when compared to the first day after surgery (2.77 mm; C.I. 2.14–3.39) (*p* = 0.03).

### 3.4. Analgesic Consumption

**Figure 5:**
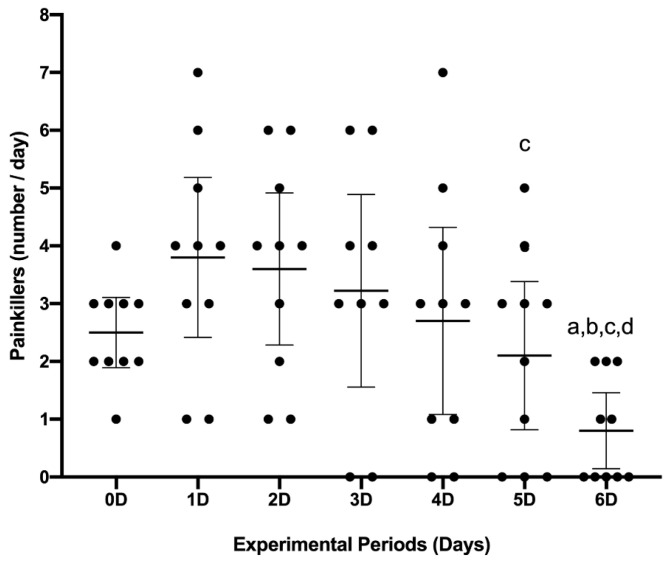
Evaluation of analgesic consumption (pills a day). The graph expresses the mean and confidence interval through the points (*n* = 10) on days 0, 1, 2, 3, 4, 5, and 6. Comparison between experimental periods identifies a significant difference represented by the letters a (≠0 Day), b (≠1 Day), c (≠2 Day), and d (≠3 Day). Tukey’s test of multiple (mixed effect analysis) and paired comparisons (*p* < 0.05).

Analgesic consumption (tablets a day) was evaluated on days 0, 1, 2, 3, 4, 5, and 6 (0.08; CI 0.14–1.45). There was a significant reduction in analgesic consumption between the groups Day 0 (2.50; C.I. 1.89–3.1), Day 1 (3.80; C.I. 2.41–5.18), Day 2 (3.60; C.I. 2.28–4.91), and Day 3 (3.22; C.I. 1.55–4.88) (*p* < 0.05). The reduction was also observed on Day 5 (2.10; I.C. 0.81–3.38) when compared to Day 2 (*p* = 0.04) (Figure 5).

### 3.5. Biochemical Analysis

A comparison was made between the ability of L-PRF and Alb-PRF membranes produced from donors to release tissue repair and inflammatory mediators through multiparametric in vitro assessment, as shown in Table 1. After 7 days of incubation, L-PRF presented an increased release of the cytokines GM-CSF, IL-1β, IL-6, TNFα, IFNy, IL-8, IL-15, IL-4, RANTES, MIP-1a, and MCP-1 (*p* < 0.05). Alb-PRF presented an increased release of the growth factor PDGF (*p* = 0.038).

**Figure 6:**
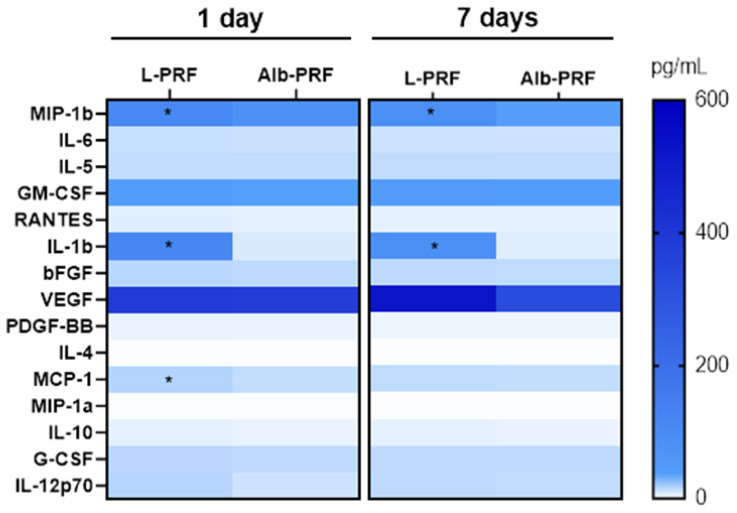
Heatmap of the variation of concentrations of different analytes identified in the surface of the surgical sites implanted with L-PRF or Alb-PRF, collected by swab after 1 or 7 days after surgery. (*) Values were statistically different between such experimental groups (*p* < 0.05).

Regarding the release of inflammatory mediators in the surgical sites, Figure 6 shows a heatmap of the surface quantification of several detectable molecules on the first and seventh day after surgery. It is possible to observe that very similar levels were detected for the main growth factors investigated (PDGF, bFGF, GM-CSF, and G-CSF) in both experimental groups. While VEGF had a higher mean concentration in the L-PRF group (525 pg/mL versus 389 for Alb-PRF), there was no significant statistical difference between both experimental sites (*p* > 0.05).

While a similar pattern was observed for several proinflammatory (IL-6, IL-5, and IL-12) and anti-inflammatory (Il-4 and IL-10) cytokines, L-PRF sites presented significantly higher levels (*p* < 0.05) of the proinflammatory cytokine IL-1β, approximately 20-fold higher than Alb-PRF, at both 1 and 7 days, and the chemokines MIP-1b, approximately three times higher than Alb-PRF, and MCP-1, which was slightly higher only at day one (11.19 pg/mL versus 8.8 pg/mL for Alb-PRF). The cytokines IL-13, IL-15, IL-10, IL-17, IL-8, IFN-γ, IL-1RA, and TNFα were not detectable in most samples and, therefore, not analyzed.

A correlation analysis was performed between each detected cytokine, and the main clinical outcomes were investigated both one day (Table 2) and seven days (Table 3) after surgery. The analysis identified a few mediators that were strongly correlated with the observed levels of clinical outcomes observed in the participants. In the L-PRF surgical sites, the levels of IL-1β at day one presented a correlation with the presence of trismus, while the report of pain (VAS at 0 and 3 days) was positively correlated to the levels of this cytokine at day seven (*p* < 0.05, Rho > 0.7). The IL-4 levels also presented a direct correlation with swelling at seven days (Rho = 0.8667, *p* < 0.05) and pain (VAS at three and five days, *p* < 0.05, Rho > 0.7) for L-PRF sites, and with initial pain (VAS at day zero) in Alb-PRF sites (Rho = 0.8359, *p* < 0.05).

In the L-PRF sites, the growth factor GM-CSF was positively correlated with trismus observed at both 1 and 7 days (*p* < 0.05, Rho > 0.7). On the other hand, in the Alb-PRF sites, RANTES levels detected on day seven were negatively correlated with pain (VAS at two and five days), while the levels of PDGF were inversely correlated to swelling (Rho = −0.7545, *p* < 0.05).

## 4. Discussion

The surgery involving lower-third molars is recognized as one of the most frequent procedures in clinical practice [43]. Adverse effects such as pain, trismus, infection, and edema often ensue post-extraction [33]. This research was conducted with the objective of linking the concentration of cytokines and growth factors at the extracted site, implanted with a new blood concentrate, either Alb-PRF and L-PRF, to clinical findings such as pain, trismus, and swelling. Increasing evidence indicates the beneficial utilization of platelet concentrates in post-extraction sites, predominantly to enhance soft tissue healing and diminish postoperative symptoms [28,44,45].

The information concerning the concentrations of growth factors and pro- and anti-inflammatory delivered cytokines on the surgical sites implanted with blood concentrates remains limited and contentious. Prior systematic reviews have assessed and compared L-PRF, PRGF, and PRP with natural healing [45,46,47]. This research provides a first-time comparison between denatured plasma combined with platelet-rich fibrin (Alb-PRF) and L-PRF in dental sockets from both clinical and in vitro perspectives. The relation between clinical parameters and analytes from the surgical site could be tied to the different cytokine profiles detected in Alb-PRF and L-PRF. The enhanced expression profile of pro-inflammatory molecules can be seen in the measurements of eluates from L-PRF membranes, both in vitro and in samples collected from surgical sites.

Despite the discussion related to the reactions provided by the cytokines evaluated in the present research, the use of autologous blood concentrates, especially L-PRF, has been discussed for its potential as a drug delivery system, using either the liquid portion or the clot [23] as the biomaterials used in the present study. Recent in vitro research has loaded the L-PRF clot with antimicrobial drugs to reduce postoperative infection risk, with promising results that underscore the utility of an autologous biomaterial for infection prevention. Although one recent study evaluating L-PRF’s antimicrobial potential showed the drug delivery system holds promise [48], L-PRF alone exhibited enough antimicrobial effect without additional drugs [49]. In this study, it was not possible to evaluate this antimicrobial effect due to postoperative antibacterial drug use following third molar extractions. Importantly, none of the subjects in the present research had postoperative infections.

Correlation has been identified between trismus (day 1) and pain (day 7) parameters with IL-1β levels, noticeable in sites that received L-PRF. This correlation can be grounded on the pro-inflammatory action of this cytokine. Alongside direct participation in inflammation orchestration, IL-1β plays a vital role in initiating nociceptive events [50]. GM-CSF, another pro-inflammatory cytokine, exhibited a correlation with trismus in L-PRF surgical sites (days 1 and 7), data that can be validated by the nature of its roles in the inflammatory process.

GM-CSF participates in the proliferation and differentiation processes of hematopoietic cells, specifically macrophages and granulocytes. During inflammation, various cells can generate GM-CSF, primarily tissue-residing cells and T and B lymphocytes. This cytokine contributes to the survival, adhesion, and traffic of neutrophils, and it boosts the antimicrobial functions of macrophages through enhanced phagocytosis and the generation of reactive oxygen species [51]. However, its elevated expression is linked to degenerative diseases such as rheumatoid osteoarthritis and spondyloarthritis [52]. Even though a correlation was noted between trismus and sites with L-PRF, no significant variations were observed in the dosage of GM-CSF between sites with L-PRF and Alb-PRF.

RANTES, defined in literature as a pro-inflammatory cytokine, primarily recruits and activates leukocytes, monocytes, granulocytes, and dendritic cells in areas of tissue injury. It also contributes to angiogenesis events and potentially affects the differentiation of osteoblasts [53]. An overexpression of RANTES was connected to the initiation of atypical facial pain and trigeminal neuralgia, which contrasts with the negative correlation found between Alb-PRF and RANTES (day 7).

Interleukin 4 (IL-4) is a versatile multipotential cytokine secreted by mast cells, eosinophils, basophils, and Th2 cells [54]. It is widely documented for its anti-inflammatory properties. Nonetheless, some evidence suggests a role of IL-4 in instigating inflammatory conditions such as dermatitis, asthma, and Kawasaki disease. The correlation between IL-4 and pain (Alb-PRF day 1) and swelling (L-PRF day 7) could be linked to this cytokine’s propensity to increase vascular permeability, contributing to extracellular fluid accumulation and consequent edema formation [55].

However, a comprehensive understanding of the correlations between specific analytes and symptomatology necessitates the consideration of these cytokines’ individual actions and the expression of the interaction of different substances and the inflammation microenvironment [56].

In past studies, the use of L-PRF has been underlined as a valid method in promoting and accelerating soft and hard tissue regeneration due to its effects on wound healing improvement, pain reduction, and bone density increase [28,44,45,46]. In this research, a significant difference in the Alb-PRF group from day 1 to day 7 for swelling was observed. These findings are not widely accepted in literature due to variations in the third molar position and surgical trauma during the operation, which directly impact postoperative pain, swelling, and trismus. It is important to note that there are other ways to measure swelling, such as using 3D images [34]. However, the Gabka and Matsumara method [41] for evaluating swelling is a straightforward and dependable way to assess inflammation and swelling in oral and facial tissue injuries. This method has been used in many recent studies [28,42,57,58,59] and is cost-effective and time-efficient. As a result, the authors have chosen to use this method in their research.

In the present study, a selection was made for a uniform set of lower third molars regarding location, type, extraction cause, adoption of minimally traumatic extraction procedures, precautions about aseptic chain during surgeries, and postoperative hygiene care instructions in order to reduce study bias.

Concerning the time points selected for outcome measurements, a period of 1 and 7 weeks for evaluating pain, swelling, and trismus appears reasonable. It has been shown that a few weeks are sufficient to achieve complete wound closure or at least complete re-epithelization, even if a second intention of soft tissue healing occurs [28,60].

As the first study evaluating the Alb-PRF using third molar extraction as a clinical model, there are limitations worth noting. The sample size of 10 participants, though adequately powered to detect differences in the primary outcomes, is relatively small. More extensive cohort studies would help validate these initial findings. While surgical protocols have been standardized, it is essential to consider that variations in trauma between subjects could potentially introduce complex factors that may need to be taken into account. Although the study did not aim to do so, a longer follow-up could provide further insights into the healing process. Nevertheless, within the parameters of this clinical trial, Alb-PRF demonstrated promising results for dental socket treatment after tooth extraction. Further research on its potential across various surgical applications is warranted.

## 5. Conclusions

While there was no difference between L-PRF and Alb-PRF in the reduction in surgical pain after mandibular third molar extractions, Alb-PRF demonstrated a significant reduction in edema after seven days. L-PRF induced a significantly higher release of pro-inflammatory cytokines when compared to Alb-PRF. Correlations were identified between different cytokines and growth factors and post-extraction symptoms. These findings suggest promising results for Alb-PRF in reducing post-surgery edema and underscore the role of cytokines and growth factors on the potential clinical applications of blood platelet concentrates in dental and broader surgical procedures.

## Figures and Tables

**Table 1:** In vitro release of biological mediators in culture media by L-PRF and Alb-PRF membranes (*n* = 5) after seven days of incubation.

Analyte	Alb-PRF	L-PRF
VEGF	810 ± 153	1104 ± 298
PDGF-BB	551 ± 39	426 ± 24 *
bFGF	28 ± 6	27 ± 12
G-CSF	381 ± 58	278 ± 74
GM-CSF	12 ± 6	57 ± 22 *
IL-1β	2 ± 2	162,7 ± 78 *
IL-6	268 ± 45	4564 ± 946 *
TNFα	39 ± 9	98 ± 24 *
IFNy	85 ± 29	246 ± 37 *
IL-8	6954 ± 782	120,589 ± 756 *
IL-13	2 ± 1.4	1.9 ± 0.8
IL-15	12 ± 8.9	96 ± 26 *
IL-7	0.46 ± 0.46	0.634 ± 0.46
IL-12p70	5 ± 4	9 ± 2
IL-17A	29 ± 8	37 ± 7
IL-9	4 ± 2	5 ± 0.1
IL-5	0.31 ± 0.1	0.3 ± 0.38
IL-2	2 ± 1	3 ± 2
IL-1RA	267 ± 70	262 ± 64
IL-4	0.9 ± 0	8.7 ± 0.7 *
IL-10	5 ± 4	19 ± 13
RANTES	593 ± 55	1084 ± 162 *
Eotaxin	133 ± 3	136 ± 9
IP-10	20 ± 7	49 ± 22
MIP-1b	170 ± 57	155 ± 59
MIP-1a	0 ± 0	39 ± 5 *
MCP-1	356 ± 22	155 ± 38 *

(*) Values were statistically different between such experimental groups (*p* < 0.05).

**Table 2:** Analysis of the correlation between pain/tissue healing indicators and the surface concentration of cytokine and growth factors on each side of the participants on day one after surgery.

	MIP-1b	IL-6	IL-5	GM-CSF	Rantes	IL-1b	bFGF	VEGF	PDGF	IL-4	MCP-1	MIP-1a	IL-10	G-CSF	IL-12
**L-PRF**															
VAS 0	0.0741	0.0124	0.1125	0.3488	0.1243	0.1359	0.2347	0.2718	0.1367	0.0124	0.1989	−0.1359	0.1359	0.0000	0.1235
VAS 1D	0.3706	0.2594	−0.4751	−0.1684	0.0621	−0.1853	−0.3336	−0.1482	−0.2610	−0.2983	−0.3356	−0.2594	−0.1853	−0.2224	0.0000
VAS 2D	0.7856	0.2578	0.1180	0.4865	0.6298	0.7365 *	0.6997	0.6138	0.4446	0.0617	0.7039	0.7243	−0.7365	0.6752	0.6506
VAS 3D	0.2546	0.3516	−0.1472	−0.0546	0.7013	0.4607	0.3758	0.3031	0.1037	−0.1646	0.3964	0.2546	0.4607	0.3395	0.5819
VAS 4D	0.1149	0.1916	−0.4459	−0.0561	0.3854	0.3448	0.1532	0.3065	0.0064	−0.2955	0.1542	0.0511	0.3448	0.1660	0.4980
VAS 5D	0.1853	0.2347	−0.0938	−0.3368	0.6649	0.3459	0.2965	0.0988	0.0249	−0.1802	0.3294	0.2471	0.3459	0.2965	0.4200
VAS 6D	0.4637	0.1364	0.0828	0.0591	0.7683	0.4637	0.5455	0.2455	0.1921	−0.1921	0.5488	0.4364	0.4637	0.4364	0.3546
Swelling 1D	0.2275	0.3713	0.6606	−0.1802	0.5904	0.2275	0.4311	−0.0240	0.3313	0.4096	0.4458	0.3353	0.2275	0.3713	0.1078
Swelling 7D	0.4458	0.7470	0.6585	0.5637	0.3212	0.5543	0.5302	0.5543	0.6303	0.8667 *	0.5515	0.4820	0.5543	0.5543	0.6627
Trismus 1D	0.3615	0.5784	0.3415	0.8469 *	0.0667	0.4097	0.3856	0.6627	0.5576	0.5758	0.3455	0.2410	0.4097	0.3253	0.4579
Trismus 7D	0.3615	0.5784	0.3415	0.8469 *	0.0667	0.4097	0.3856	0.6627	0.5576	0.5758	0.3455	0.2410	0.4097	0.3253	0.4579
**Alb-PRF**															
VAS 0	0.0507	0.1212	0.7148	0.0761	−0.4019	0.1142	0.2297	0.2029	0.4059	0.8230 *	0.3828	0.2599	0.4820	0.6343	0.2029
VAS 1D	−0.6789	−0.3598	−0.2857	−0.3273	−0.1403	−0.5577	−0.4452	−0.2667	−0.1576	−0.4147	−0.0793	−0.4969	−0.1818	0.2063	−0.3637
VAS 2D	−0.4051	−0.3087	0.3145	0.0859	−0.1111	−0.2455	−0.1173	0.2701	0.0982	0.0185	0.2408	−0.2138	0.0491	0.2785	−0.0737
VAS 3D	−0.3953	−0.3915	0.0380	−0.1977	−0.0932	−0.2841	−0.0994	0.0741	−0.1730	0.0311	−0.0994	−0.2279	0.0494	0.0955	−0.0247
VAS 4D	0.1091	−0.0061	−0.4596	0.1940	0.5000	0.2182	0.2500	0.4243	−0.2303	−0.4452	−0.1525	0.1242	0.0849	−0.2938	0.3273
VAS 5D	−0.4243	−0.2927	−0.1491	−0.1455	−0.0549	−0.3031	−0.1037	0.0121	−0.0364	0.0000	0.0061	−0.2236	0.0727	0.2875	−0.0242
VAS 6D	0.0261	−0.0262	0.2673	0.0913	0.0262	0.1174	0.3214	0.3651	0.2739	0.5051	0.3214	0.2138	0.4825	0.4169	0.3651
Swelling 1D	0.1905	−0.0120	0.1220	−0.0952	−0.1317	0.0952	0.1677	−0.0952	−0.0238	0.5150	−0.1437	0.1464	0.0952	−0.0123	0.1429
Swelling7D	0.1429	0.3593	−0.3660	0.0238	0.2515	0.0952	0.2156	−0.1190	0.6190	0.2515	0.5749	0.2196	0.4286	0.6383	0.2381
Trismus 1D	0.0507	0.1212	0.7148	0.0761	−0.4019	0.1142	0.2297	0.2029	0.4059	0.8230	0.3828	0.2599	0.4820	0.6343	0.2029
Trismus 7D	−0.6789	−0.3598	−0.2857	−0.3273	−0.1403	−0.5577	−0.4452	−0.2667	−0.1576	−0.4147	−0.0793	−0.4969	−0.1818	0.2063	−0.3637

(*) Values were statistically different between such experimental groups (*p* < 0.05).

**Table 3:** Analysis of correlation between pain/tissue healing indicators and the surface concentration of cytokine and growth factors on each side of the participants, seven days after surgery.

	MIP-1b	IL-6	IL-5	GM-CSF	Rantes	IL-1b	bFGF	VEGF	PDGF	IL-4	MCP-1	MIP-1a	IL-10	G-CSF	IL-12
**L-PRF**															
VAS 0	0.2648	0.1493	0.4352	−0.1053	0.5971	0.8533 *	0.8533	0.5002	−0.0294	0.2239	0.8359 *	−0.0883	0.7945	−0.1045	0.6473
VAS 1D	0.1765	0.0896	−0.7833	0.7105	−0.8508	−0.2648	−0.2648	0.3531	0.4414	0.0000	−0.2239	0.5296	−0.1765	0.5374	0.0883
VAS 2D	0.3531	0.6717	0.7833	0.0811	0.1343	0.6179	0.6179	0.2648	0.1765	0.6269	0.5822	0.3531	0.5296	0.3582	0.2648
VAS 3D	0.4638	0.6176	0.2572	0.7632	0.1471	0.8117 *	0.8117	0.7537	0.6377	0.8676 *	0.7353	0.7537	0.6377	0.5441	0.5508
VAS 4D	0.1518	0.3388	0.1796	0.7895	−0.0924	0.6375	0.6375	0.7590	0.4554	0.6776	0.5236	0.6983	0.3947	0.3696	0.3339
VAS 5D	0.4414	0.6717	0.2611	0.6489	0.1791	0.6179	0.6179	0.5296	0.7062	0.9404 *	0.5374	0.7945	0.4414	0.5822	0.3531
VAS 6D	0.4554	0.7701	0.7184	0.1579	0.2772	0.6983	0.6983	0.3339	0.3947	0.8317	0.6468	0.5161	0.5768	0.4620	0.3339
Swelling 1D	0.8117	0.6765	0.0000	−0.5526	0.5735	0.3189	0.3189	−0.0580	0.5508	0.4559	0.4265	0.1739	0.5218	0.4853	0.5218
Swelling 7D	0.6179	0.1941	−0.3482	−0.3785	0.5523	0.4119	0.4119	0.2354	0.3237	0.0448	0.5224	−0.0883	0.6179	0.1642	0.7356
Trismus 1D	0.1471	−0.2090	−0.3482	−0.0263	−0.2239	0.1177	0.1177	0.2648	−0.2354	−0.5224	0.2239	−0.3237	0.3237	−0.0746	0.4414
Trismus 7D	0.1471	−0.2090	−0.3482	−0.0263	−0.2239	0.1177	0.1177	0.2648	−0.2354	−0.5224	0.2239	−0.3237	0.3237	−0.0746	0.4414
**Alb-PRF**															
VAS 0	0.0381	0.8371	0.2680	0.3947	0.1649	0.7357	0.6469	0.6596	0.2361	0.5263	0.8677	0.4275	0.6571	0.7401	0.6508
VAS 1D	−0.0121	−0.3395	0.1098	−0.3479	−0.7638	−0.4243	−0.3516	0.4122	−0.0610	−0.3926	−0.2012	−0.2988	0.2805	−0.2622	0.2805
VAS 2D	0.3805	0.2578	0.4199	0.2648	−0.3805 *	0.1350	0.1719	0.5524	−0.1173	0.2858	0.4755	−0.2470	0.7410	0.2038	0.7039
VAS 3D	−0.0124	0.3212	0.7830	−0.0883	−0.6054	0.1482	0.2100	0.5930	0.6152	0.3001	0.2361	0.3915	0.4039	0.3977	0.3480
VAS 4D	0.1091	−0.0364	0.4756	−0.0294	−0.4364	−0.1940	0.1818	0.0364	0.5366	0.3374	−0.3171	0.0610	0.0061	0.2012	−0.1159
VAS 5D	0.0242	0.1697	0.6098	−0.0883	−0.7759 *	0.0727	0.1576	0.7274	0.5854	0.1288	0.1403	0.3476	0.4391	0.2866	0.4025
VAS 6D	0.1826	0.7303	0.7347	0.3086	−0.2608	0.4564	0.5477	0.7303	0.6888	0.5544	0.5969	0.5510	0.6888	0.7806	0.6428
Swelling 1D	−0.2857	0.4286	0.4192	0.0857	0.0714	0.5238	0.3571	0.1905	−0.7545 *	0.3012	0.2275	0.9341	−0.1916	0.4311	−0.1796
Swelling 7D	0.1905	−0.0714	−0.2755	−0.3143	−0.1667	−0.2619	−0.2143	0.3571	0.0838	−0.5061	−0.0599	0.0479	0.3234	0.0000	0.4192
Trismus 1D	0.2771	−0.2048	−0.0364	0.1449	−0.1084	−0.3133	0.0602	−0.2289	−0.2546	0.2378	−0.2606	−0.6364	0.0909	−0.0788	−0.0242
Trismus 7D	0.2771	−0.2048	−0.0364	0.1449	−0.1084	−0.3133	0.0602	−0.2289	−0.2546	0.2378	−0.2606	−0.6364	0.0909	−0.0788	−0.0242

(*) Values were statistically different between such experimental groups (*p* < 0.05).

## Data Availability

No new data were created.
